# Determining farm surface porcine reproductive and respiratory syndrome virus (PRRSV) contamination through viability RT-qPCR

**DOI:** 10.1371/journal.pone.0344714

**Published:** 2026-03-13

**Authors:** Claudio Marcello Melini, Amanda Palowski, Declan C. Schroeder, Cesar A. Corzo

**Affiliations:** Department of Veterinary Population Medicine, University of Minnesota, Saint Paul, Minnesota, United States of America; University of Nicolaus Copernicus in Torun, POLAND

## Abstract

Porcine reproductive and respiratory syndrome (PRRS) continues to be a major threat to U.S. swine industry, as a substantial number of herds become positive and can pose a risk to other nearby farms, especially in post weaning farms as multiple of them may be overseen by one worker. Personnel moving between farms without adequate biosecurity measures, may play a role in viral spread acting as a fomite. The ability to detect and distinguish between the free PRRS virus (PRRSV) genomic RNA vs its genome found in a viable virus form on frequently touched surfaces in growing pig farms was assess in this study. Ten PRRSV positive growing pig farms in the Midwestern U.S. were visited to collect 20 environmental surface samples and eight oral fluids from each one. Environmental samples were analyzed using standard RT-qPCR and viability RT-qPCR, while oral fluids were assessed using the VetMAX™ PRRSV EU & NA v3.0 kit. The virus’ RNA was commonly detected on metal and plastic (non-porous) surfaces (e.g., pig pen top rail, mortality handling equipment’s handle) from all farms, with 80 out of 200 environmental samples testing positive. Viable virus was detected in 48 samples across six farms, with non-porous materials testing positive more frequently. A generalized linear mixed effect model suggested a negative association (OR = 0.005; 95% CI 0.00, 6.82; p-value = 0.18) between the proportion of positive oral fluids and detecting viable virus from sampled surfaces. Agreement between the detection of RNA and viable PRRSV from surface samples using Cohen’s kappa yielded perfect agreement (κ = 1.00) from doorknobs of different locations, to low agreement (κ = 0.29) in the floor of a specific area, among others. These results indicate the presence of viable virus on surfaces that are frequently touched by the farm’s personnel. This study highlights the importance of biosecurity measures applied to the personnel and their potential role of environmental contamination and PRRSV dissemination. The use of viability RT-qPCR to detect viable PRRSV offers a practical tool in field settings to improve biosecurity protocols to reduce indirect transmission of PRRSV in swine production systems.

## Introduction

Porcine reproductive and respiratory syndrome (PRRS) caused by the virus named after the disease, PRRSV, remains a costly disease in the U.S. [1–4]. Annually, 20–30% of the breeding herds enrolled in the Morrison Swine Health Monitoring Project (MSHMP) report a PRRS outbreak [5]. Furthermore, about 30% of the breeding herds in the MSHMP persist in category 1 of the American Association of Swine Veterinarians (AASV) PRRSV breeding herd guideline classification system [6,7], which indicates that these herds are weaning PRRSV positive pigs. Recently weaned PRRSV positive pigs are usually raised in nursery farms which means that multiple growing pig sites are being filled with infected pigs, ultimately posing a risk to other populations in the region. The regional transmission phenomenon is exemplified by a report showing that 44% of the growing pig groups weaned from herds weaning virus free pigs become PRRSV positive before reaching market [8]. Another study found that 24% of the sites housing near-to-market pigs were RT-PCR positive, reinforcing the hypothesis that PRRS-infected growing pig populations pose a risk to neighboring farms [9]. A lingering question is why and how these groups of growing pigs become infected. Biosecurity measures and compliance play a crucial role [10], especially in an industry where specific designated individuals may directly and indirectly interact with large populations of growing pigs across different sites in one day. These individuals move from site to site, ensuring pigs have access to feed, water, heat, and proper air quality. In addition, caretakers are responsible for ensuring that pigs are treated in a timely manner, and those that have died are removed from the barn. As most growing pig sites lack shower-in/shower-out facilities [11], there is an opportunity for contaminated personnel to act as carriers and play a role in viral transmission across and between sites of different PRRS statuses. Identifying viable PRRSV on frequently touched surfaces may offer additional insight into the potential for indirect transmission and could help reinforce the importance of biocontainment, biomanagement, and bioexclusion protocols.

Recently, we documented through using the Veterinary Diagnostic Laboratory PRRSV RT-PCR platform that PRRSV RNA could be found on interior and exterior pig farm surfaces of farms housing infected pigs [12], leaving open the possibility that these positive samples could be a source of viable virus. Experimental data also suggests that PRRSV can survive *ex vivo* for hours and days, emphasizing the potential for viable virus dissemination [13,14]. Recently, a viability quantitative PCR (V-qPCR) assay was developed for African swine fever virus (ASFV); and similarly the viability RT-qPCR for PRRSV [15] to evaluate directly whether the virus particle remains intact or viable after a specific treatment. To address whether PRRSV captured from various environments on a farm can pose a biological risk, this study aimed to 1) determine whether viable PRRSV can be detected on frequently touched surfaces by farm personnel; 2) assess whether the number of PRRSV detections from oral fluids in a population are associated with the detection of viable PRRSV on farm surfaces; and 3) explore the agreement between standard and viability RT-qPCR for monitoring PRRSV under field conditions.

## Materials and methods

### Study design

In this observational cross-sectional study, ten Midwestern U.S. conveniently selected growing pig sites from one production system were enrolled. Farm eligibility criteria included: 1) growing pig barns with at least 2,000 pigs, 2) recent infection (i.e., within 2–5 weeks of the study’s sample collection) with a wild-type PRRSV following molecular testing reports by the production system veterinarian, 3) growing pigs must have been sourced from a PRRSV naïve (AASV Category IV) sow herd (e.g., seronegative) and remained negative in the nursery barns; and 4) obtainable PRRSV sequence.

### Sample size

Based on previous environmental sampling PRRSV detection results [12], it was decided to collect 20 surface samples per farm which allowed us to successfully detect viral RNA. This sample size provided a 95% probability of detecting at least one positive surface sample when the proportion of positive samples was at least 14%. Additionally, the level of PRRSV population shedding was determined through oral fluid testing, using the University of Minnesota (UMN) veterinary diagnostic laboratory (VDL) PRRSV testing protocols. Therefore, a within-herd prevalence of at least 20% was assumed and per previous work on oral fluid sample size estimation [16,17], a set of 8 ropes were hung between pens which ultimately represented 16 pens per barn.

### Surface sampling

The list of surfaces to sample and the sampling methodology were elaborated based on a previously validated PRRSV environmental detection study [12], and in conjunction with swine veterinarians’ input (Table 1). Briefly, investigators wore a clean set of disposable coveralls (Tyvek®, Dupont™, Wilmington, DE, USA), two sets of disposable plastic boot covers, and a pair of clean nitrile gloves during sample collection. Gloves were changed between each sampling event, and hands were cleaned using disinfecting wipes to avoid potential cross-contamination. Depending on the selected surface, a 30 cm by 30 cm area or in the case of irregular surfaces (i.e., doorknob, bottle, handles) the whole surface was rubbed with a folded 26.5 cm by 20.3 cm wipe (Swiffer®, P&G, Cincinnati, OH, USA), previously moistened with 20 ml of Dubelcco’s modified eagle medium (DMEM) (Gibco, ThermoFisher Scientific, Waltham, MA, USA), and then placed in a resealable plastic bag (Ziploc®, S.C. Johnson, Racine, WI, USA). Through pressure, the expected 5–15 ml of liquid was extracted from the wipe and poured into a 20 mL sterile tube (Corning Falcon®, Corning Science Mexico S.A. de C.V., Tamaulipas, MX). Samples were labeled according to a unique farm and surface identifier, and then refrigerated for transportation to the UMN. From the veterinary laboratory health reports of each farm, PRRSV open reading frame 5 (ORF5) gene sequence results were obtained and used to identify which strain was present at the time of the outbreak. In the case that this information was not available, oral fluids that were collected for the study were used to obtain the sequences following the UMN VDL PRRS ORF5 sequencing protocol, and classified using the method described by [18–20] using the application PRRSLoom [21] and its version before the March 6^th^ 2025 update.

**Table 1:** Location, number of samples, surface material, and porosity classification of sampled surfaces in porcine reproductive and respiratory syndrome positive growing pig farms selected for the study.

Surface sample location	Number of collected samples	Surface material	Porosity
Main entry floor close to the bench (or separation of clean and dirty area)	1	Concrete/Vinyl	Porous/Non-porous
Main entry door’s exterior doorknob	1	Metal	Non-porous
Main entry door’s interior doorknob	1	Metal	Non-porous
Main entry’s interior floor	1	Concrete/Vinyl	Porous/Non-porous
Floor before entering pig space room	1	Concrete	Porous
Floor of the pig space room’s interior	1	Concrete	Porous
Pig space room door’s exterior doorknob	1	Metal	Non-porous
Pig space room door’s interior doorknob (pig side)	1	Metal	Non-porous
Mortality handling equipment’s handle (or boots/snare handle)	2	Rubber/Metal	Non-porous/Non-porous
Pig pen top rail	2	Metal	Non-porous
Sorting paddle/board’s handle	2	Plastic	Non-porous
Used injectable bottle (or applicator)	2	Glass/Plastic	Non-porous/Non-porous
Loading chute/mortality removal exterior doorknob (or floor)	1	Metal/Wood	Non-porous/Porous
Loading chute/mortality removal interior doorknob (or floor)	1	Metal/Wood	Non-porous/Porous
Exhaust fan cone	2	Plastic	Non-porous

### Oral fluid sampling

At each farm, a set of eight cotton ropes were hung following recommendations from [17] and [16]. Briefly, depending on the number of pens in the room, the ropes were hung for 45–60 minutes, and were distributed evenly on both sides of the barn with each rope being placed between two pens representing approximately 60 pigs, at a reachable height for the pigs. A set of clean nitrile gloves were used when placing and retrieving the ropes. Gloves were changed, and disinfection wipes to clean investigator’s hands were also used in between sampling ropes. After collecting each rope, these were placed inside a resealable plastic bag (Ziploc®, S.C. Johnson, Racine, WI, USA). The liquid was extracted by squeezing the rope from the outside of the bag and then pouring it into a 20 mL sterile tube (Corning Falcon®, Corning Science Mexico S.A. de C.V., Tamaulipas, MX) labeled according to placement and farm. The sampling methodology was approved by the UMN’s Institutional Animal Care and Use Committee (IACUC) (Protocol #2305-41068A).

### Sample processing

The samples were centrifuged at 1500g at 4°C for 10 minutes. Four 600 µL aliquots were obtained for each environmental sample, and two 1000 µL aliquots from oral fluids and then stored at -80C.

### Environmental sample testing

#### Standard-RT-qPCR (S-RT-qPCR).

As previously described in [15], 200 µL aliquot units of sample was used for RNA extraction (NucleoMag® VET, Takara Bio USA., Inc., Mountain View, CA, USA) to be used as a template for RT-PCR. The final 50 µL elution volumes of RNA were stored at -80^o^C until S-RT-qPCR analysis was conducted using 1uL of RNA in 19uL of VetMAX™-Plus Multiplex One Step RT-PCR kit (ThermoFisher Scientific Inc., Waltham, MA, USA) following the manufacturer’s instructions using the following conditions: 10 minutes at 48 °C, and 10 minutes at 95 °C followed by 40 cycles of 15 seconds at 95 °C, and 60 seconds at 60 °C. All RT-qPCR assays were conducted using Roto-gene Q Real-Time PCR (Qiagen, CA, USA). Samples were considered positive if the cycle threshold (Ct) value was below 40.

#### Viability-RT-qPCR diagnostic (V-RT-qPCR).

A second duplicate 200 µL aliquot of the same sample used for the S-RT-qPCR assays were used for the V-RT-qPCR by adding PMAxx dye (Biotium Inc, CA, USA) to obtain a 25 µM final concentration (Balestreri et al.2024). All samples were incubated in the dark at room temperature for 10 minutes on a rocker for optimal mixing. The PMAxx treated samples were then exposed to light for 30 minutes using a PMA-Lite device (Biotium Inc, CA, USA) to cross-link PMAxx dye to the RNA (free RNA or RNA from within broken/damaged PRRS viruses). All samples were then used for RNA extraction (NucleoMag® VET, Takara Bio USA., Inc., Mountain View, CA, USA) to be used as a template for RT-PCR. The final 50 µL elution volumes of samples were stored at -80^o^C until V-RT-qPCR analysis was conducted using 1uL of RNA in 19uL of VetMAX™-Plus Multiplex One Step RT-PCR kit (ThermoFisher Scientific Inc., Waltham, MA, USA) following the manufacturer’s instructions using the following conditions: 10 minutes at 48 ° C, and 10 minutes at 95 ° C followed by 40 cycles of 15 seconds at 95 ° C, and 60 seconds at 60 ° C. All RT-qPCR assays were conducted using Roto-gene Q Real-Time PCR (Qiagen, CA, USA). Samples were considered positive if the Ct value was below 40.

### Oral fluids testing

One aliquot of oral fluid sample was submitted to the UMN VDL for RNA simple extraction (MagMax™ CORE Nucleic Acid Purification Kit, ThermoFisher Scientific Inc., Waltham, MA, USA) by loading the beads and protease K together at a 30 µL volume, and then adding 200 µL of the sample, and 700 µL of lysis/Xeno RNA mix, and PRRSV RT-PCR by using VetMAX™ PRRSV EU & NA v3.0 kit (ThermoFisher Scientific Inc., Waltham, MA, USA) following the manufacturer’s instructions. In accordance with the UMN VDL’s reporting criteria, samples were considered positive if the Ct value was below 40.

### Statistical analyses

Statistical analyses were performed using R software [22], and the packages base [22], lme4 [23], psych [24], and vcd [25]. Descriptive statistics were used to summarize the results. The association between PRRSV detection in oral fluids and viable virus on surfaces was assessed using a generalized linear mixed model (GLMM). Using the number of detections of viable virus on surfaces as the outcome, the number of detections from oral fluids as the predictor, and each farm as a random effect. Additionally, the distribution was inputted as binomial, and the link function as logit due to the outcome being a proportion.

Agreement of the detections of PRRSV on surfaces between standard and viability RT-qPCR was performed using Cohen’s Kappa [26–28], considering the results as positive if the Ct value was below 40, an negative if it was equal to 40. The 95% confidence interval (CI), p-value of the analysis, and global agreement were also reported.

## Results

Surface sampling in the 10 enrolled PRRSV positive growing pig farms took place between June and July (summer) of 2024. Farms visits were divided into four groups of two to three farms to comply with production company biosecurity measures. All samples were successfully collected; however, all but two ropes from different farms were not collected as they were lost in the collection process.

Two ORF5 gene sequences were obtained from the system’s veterinarian corresponding to two farms, and oral fluids with the lowest Ct values from eight farms were submitted to the UMN VDL for ORF5 gene sequencing to complete the PRRSV sequence information. Sequence results showed eight farms had an L1C.5 variant, one farm had an L1A-unclassified, and one farm had a partial sequence with a result of L1C-unclassified. Results are summarized in Tables 2 and 3.

**Table 2:** Proportion of positive surface samples by standard-RT-qPCR and viability-RT-qPCR, oral fluids by RT-PCR per farm, state location, biosecurity feature, and porcine reproductive and respiratory syndrome virus classification.

Farm identification	State	Presence of shower	PRRSV sublineage	Surface S-RT-qPCR positives/ total number of samples (%)	Surface V-RT-qPCR positives total number of samples (%)	Oral fluid RT-PCR positives total number of samples (%)
1	Minnesota	No	1C.5	6/20 (30%)	0/20 (0%)	8/8 (100%)
2	Iowa	No	1C.5	4/20 (20%)	1/20 (5%)	6/8 (75%)
3	Iowa	No	1C.5	5/20 (25%)	0/20 (0%)	7/7 (100%)
4	Minnesota	Yes	1C.5	15/20 (75%)	14/20 (70%)	5/8 (63%)
5	Minnesota	No	1C.5	10/20 (50%)	8/20 (40%)	7/8 (88%)
6	Minnesota	No	1C.5	2/20 (10%)	0/20 (0%)	7/7 (100%)
7	Iowa	Yes	1C.5	1/20 (5%)	0/20 (0%)	7/8 (88%)
8	Iowa	No	1C.5	5/20 (25%)	2/20 (10%)	4/8 (50%)
9	Iowa	No	1A-unclassified	15/20 (75%)	12/20 (60%)	8/8 (100%)
10	Iowa	No	1C-unclassified^a^	17/20 (85%)	11/20 (55%)	5/8 (63%)

^a^Assignment is with low confidence.

**Table 3:** Number of standard-RT-qPCR and viability-RT-qPCR porcine reproductive and respiratory syndrome virus positive surface samples by material and porosity classification.

Surface material (porosity) classification	RT-qPCR			
	Standard		Viability	
	Positive/total number of samples (%)	Median (Q1, Q3) Ct value	Positive/ total number of samples (%)	Median (Q1, Q3) Ct value
Metal (non-porous)	36/85 (42.4%)	29.2 (28.4, 30.5)	23/85 (27.1%)	28.2 (28.0, 28.5)
Plastic (non-porous)	24/55 (28.2%)	29.1 (28.0, 30.9)	14/55 (16.5%)	27.9 (27.8, 28.1)
Concrete (porous)	12/33 (14.1%)	30.4 (29.7, 32.0)	5/33 (5.9%)	28.3 (27.4, 28.4)
Rubber (non-porous)	3/6 (3.5%)	30.4 (29.2, 32.1)	1/6 (1.2%)	28.2 (28.2, 28.2)
Vinyl (non-porous)	3/8 (3.5%)	27.7 (27.6, 28.1)	3/8 (3.5%)	27.6 (27.4, 27.8)
Glass (non-porous)	2/7 (2.4%)	28.2 (28.1, 28.4)	2/7 (2.4%)	27.2 (27.2, 27.2)
Wood (porous)	0/6 (0%)	- (-, -)	0/6 (0%)	- (-, -)

### Oral fluids

Detection of PRRSV RNA through RT-PCR from oral fluids was possible in all farms, positivity rate ranged between 50% and 100% per farm (Table 2). The mean PRRSV RT-PCR Ct value was 33.9, ranging from 26.4 to 36.1.

### Environmental sample testing via S-RT-qPCR and V-RT-qPCR

Overall, PRRSV was detected in all farms with 80 out of 200 samples collected with a median Ct value of 29.3, a quantile (Q) 1 of 28.2, and a Q3 of 30.8. On-farm detection of PRRSV ranged from one (5%) to 17 (85%) out of 20 samples (Table 2).

**Fig 1:**
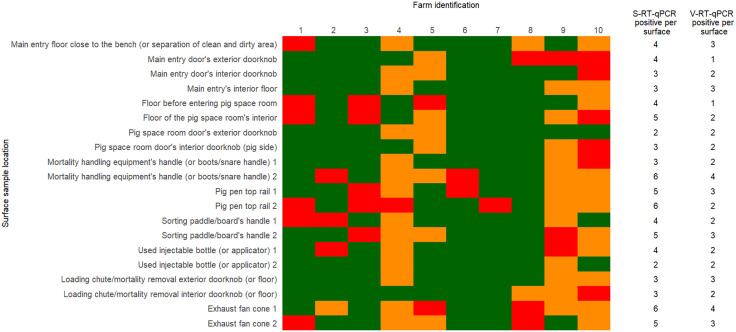
Surface standard-RT-qPCR and viability-RT-qPCR results by location and farm. Surface was either negative to both S-RT-qPCR and V-RT-qPCR (green), positive for S-RT-qPCR (red), or positive for both S-RT-qPCR and V-RT-qPCR (orange).

**Fig 2:**
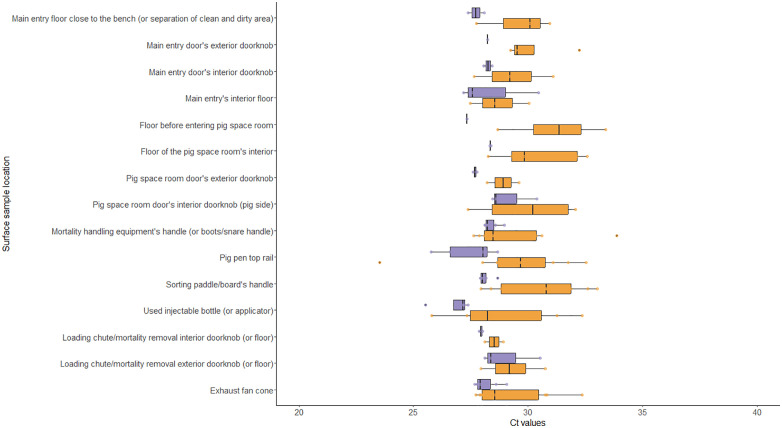
Distribution of cycle threshold value results by assay and location. Assay type was either S-RT-qPCR (orange) or V-RT-qPCR (purple).

Detection of the virus and Ct values varied according to location and surfaces, (Table 4) (Figs 1 and 2) with the “exhaust fan cone” (n = 11), “pig pen top rail” (n = 11), “mortality handling equipment’s handle” (n = 9) and “sorting paddle/board’s handle” (n = 9) being the most frequently positive surfaces.

**Table 4:** Number of porcine reproductive and respiratory syndrome virus positive standard-RT-qPCR and viability-RT-qPCR surface samples and median cycle threshold values by location.

Surface sample location	RT-qPCR			
	Standard		Viability	
	Positive/ total number of samples	Median (Q1, Q3) Ct value	Positive/ total number of samples	Median (Q1, Q3) Ct value
Main entry floor close to the bench (or separation of clean and dirty area)	4/10 (40%)	30.1 (28.9, 30.5)	3/10 (30%)	27.7 (27.5, 27.9)
Main entry door’s exterior doorknob	4/10 (40%)	29.5 (29.4, 30.3)	1/10 (10%)	28.2 (28.2, 28.2)
Main entry door’s interior doorknob	3/10 (30%)	29.2 (28.4, 30.2)	2/10 (20%)	28.3 (28.2, 28.3)
Main entry’s interior floor	3/10 (30%)	28.6 (28.0, 29.3)	3/10 (30%)	27.6 (27.4, 29.0)
Floor before entering pig space room	4/10 (40%)	31.4 (30.2, 32.3)	1/10 (10%)	27.3 (27.3, 27.3)
Floor of the pig space room’s interior	5/10 (50%)	29.9 (29.3, 32.1)	2/10 (20%)	28.4 (28.3, 28.4)
Pig space room door’s exterior doorknob	2/10 (20%)	28.9 (28.6, 29.3)	2/10 (20%)	27.7 (27.6, 27.7)
Pig space room door’s interior doorknob (pig side)	3/10 (30%)	30.2 (28.4, 31.8)	2/10 (20%)	28.6 (28.5, 29.5)
Mortality handling equipment’s handle (or boots/snare handle)	9/20 (45%)	28.5 (28.1, 30.4)	6/20 (30%)	28.2 (28.2, 28.5)
Pig pen top rail	11/20 (55%)	29.7 (28.7, 30.8)	5/20 (25%)	28.0 (26.6, 28.2)
Sorting paddle/board’s handle	9/20 (45%)	30.8 (28.8, 31.9)	5/20 (25%)	28.0 (27.9, 28.2)
Used injectable bottle (or applicator)	6/20 (30%)	28.2 (27.5, 30.6)	4/20 (20%)	27.2 (26.7, 27.2)
Loading chute/mortality removal interior doorknob (or floor)	3/10 (30%)	28.5 (28.3, 28.7)	3/10 (30%)	28.0 (27.9, 28.0)
Loading chute/mortality removal exterior doorknob (or floor)	3/10 (30%)	29.2 (28.6, 29.9)	2/10 (20%)	28.4 (28.2, 29.4)
Exhaust fan cone	11/20 (55%)	28.5 (28.0, 30.5)	7/20 (35%)	27.9 (27.8, 28.4)

**Fig 3:**
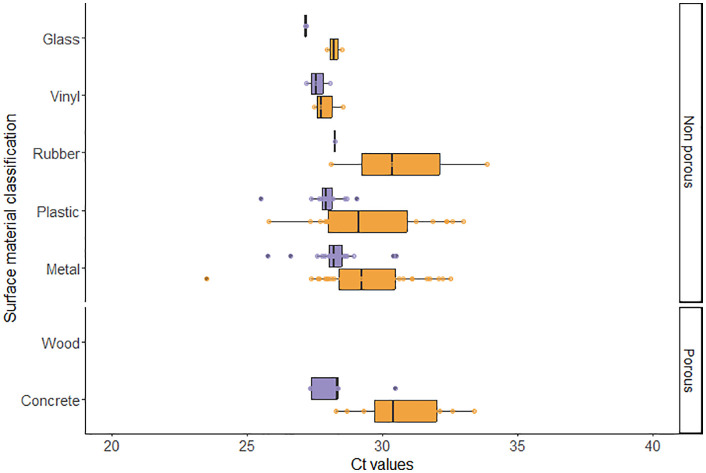
Distribution of cycle threshold value results by assay, surface material, and porosity classification. Assay type was either S-RT-qPCR (orange) or V-RT-qPCR (purple).

Regarding the type of surface material, the virus was detected more frequently in metal (n = 36), followed by plastic (n = 24), concrete (n = 12), rubber (n = 3), vinyl (n = 3), and glass (n = 2). While there were no detections on wood (Fig 3). Surfaces classified as non-porous (n = 68) had greater detection than porous surfaces (n = 12). The S-RT-qPCR’s Ct values results of each surface material are summarized in Fig 3.

Detection of viable PRRSV by V-RT-qPCR was possible in 48 out of the 200 samples collected with positives originating from six out of 10 farms (Fig 1). The number of positive samples ranged from one (5%) to 14 (70%) per farm (Table 2). Locations where viable virus was detected originated from “exhaust fan cones” (n = 7), “mortality handling equipment handle (or boots/snare handle)” (n = 6), “pig pen top rail” (n = 5), and “sorting paddle/board handle” (n = 5) (Table 4). Regarding the surface material and porosity classification of the surfaces, detections were more frequent on surface materials classified as metal (n = 23), followed by plastic (n = 14), concrete (n = 5), vinyl (n = 3), glass (n = 2), and rubber (n = 1). And non-porous surfaces (n = 43) yielding most of the positive detections (Table 3). The S-RT-qPCR’s Ct values results of each surface location, material, and porosity classification are summarized in Figs 2 and 3.

### Presence of PRRSV on surfaces in pig barns and the detection on oral fluids

The proportions of positive oral fluids were used to assess the association of detecting viable PRRSV from sampled surfaces per farm using a GLMM with a binomial distribution and a logit link function. The odds ratio (OR), 95% CI, and p-value were calculated for interpretation. The model suggested a negative association, as for every unit increase in the proportion of positive oral fluids there was a decrease in the odds of detecting viable virus on surfaces in the farms. While the model suggested a negative association between the proportion of positive oral fluids and the odds of detecting viable virus on surfaces of the barns this was statistically significant (OR = 0.005; 95% CI 0.00, 6.82; p-value = 0.18).

### Agreement between S-RT-qPCR and V-RT-qPCR PRRSV detection

Cohen’s kappa was used to explore the agreement between the detections of RNA and viable PRRSV from different surfaces in farms using S-RT-qPCR and V-RT-qPCR, respectively. Overall, the surfaces with perfect agreement (κ = 1.00) were the “main entry interior floor”, “pig space room door exterior doorknob”, and “loading chute/mortality removal interior doorknob (or floor)”. And the ones with the lowest agreement (κ = 0.29) were “main entry door exterior doorknob” and “floor before entering pig space room” (Table 5).

**Table 5:** Cohen’s kappa and global agreement of detection of porcine reproductive and respiratory syndrome between two assays.

Assay comparison	Surface sample location	Cohen’s kappa (95% CI)	Cohen’s kappa p-value	Global Agreement
S-RT-qPCR and V-RT-qPCR	Main entry floor close to the bench (or separation of clean and dirty area)	0.74 (0.26, 1.00)	0.002*	90%
	Main entry door’s exterior doorknob	0.29 (−0.19, 0.76)	0.237	70%
	Main entry door’s interior doorknob	0.74 (0.26, 1.00)	0.002*	90%
	Main entry’s interior floor	1.00 (1.00, 1.00)	0.000*	100%
	Floor before entering pig space room	0.29 (−0.19, 0.76)	0.237	70%
	Floor of the pig space room’s interior	0.40 (−0.05, 0.85)	0.085	70%
	Pig space room door’s exterior doorknob	1.00 (1.00, 1.00)	0.000*	100%
	Pig space room door’s interior doorknob (pig side)	0.78 (0.39, 1.00)	<0.001*	90%
	Mortality handling equipment’s handle (or boots/snare handle)	0.69 (0.38, 1.00)	<0.001*	85%
	Pig pen top rail	0.43 (0.11, 0.74)	0.007*	70%
	Sorting paddle/board’s handle	0.58 (0.24, 0.91)	<0.001*	80%
	Used injectable bottle (or applicator)	0.74 (0.40, 1.00)	<0.001*	90%
	Loading chute/mortality removal interior doorknob (or floor)	1.00 (1.00, 1.00)	0.000*	100%
	Loading chute/mortality removal exterior doorknob (or floor)	0.78 (0.39, 1.00)	<0.001*	90%
	Exhaust fan cone	0.61 (0.30, 0.93)	<0.001*	80%

*Statistically significant.

## Discussion

Detection of viral genetic material (DNA/RNA) on different types of surfaces in swine barns using molecular diagnostic tools such as PCR/RT-PCR is known to be possible [12,29,30]; however, little information is available regarding the viability of the detected viruses. In this study, we report the presence of viable PRRSV on frequently touched surfaces across multiple farms. While this finding highlights the potential for indirect transmission through contaminated surfaces, particularly in areas with high personnel traffic, it is important to note that the study did not establish a direct link between surface contamination and PRRSV transmission. Furthermore, the lack of a statistically significant association between oral fluid positivity and viable virus detection suggests that the role of these surfaces in active transmission remains unclear and warrants further investigation.

Viable and traces of virus genome were detected on different used injectable bottles and medicine applicators. Notably, some of the bottles’ samples had been returned to storage boxes before they were disposed of, which suggests that the virus under these environmental conditions can remain viable. Similarly, detection of viable virus on main entrance doorknobs was revealing as this finding highlights the risk of caretakers carrying virus on their hands as they either enter or leave the facility. Detecting virus on an exterior doorknob that is exposed to sunlight and other environmental conditions that could hinder virus viability was not a limitation in this case. This specific sample was collected before farm personnel arrived at the farm, suggesting that the virus may have been on that surface for multiple hours, but more importantly, it also raises the question regarding employees contaminating the doorknob the day before the sample was collected which is a biocontainment concern. The possibility of personnel involved in the contamination of surfaces (i.e., door handles, bench, etc.) even when they have been trained for the use of the bench and shower-in/-out system has been described previously [31], thereby supporting the findings of viral detection in surfaces that do not have direct contact with pigs but mostly in contact with personnel.

Although a formal assessment of the effect of surface type (i.e., porous and non-porous) was beyond the scope of this study, it provides evidence that both PRRSV RNA and viable virus particles were more frequently detected in non-porous (RNA n = 68, viable n = 43) than porous (RNA n = 12, viable n = 5) surfaces, which agrees with previous reports involving PRRSV, SARS-CoV-2, and Influenza virus [32–36]. This may be explained as porous surfaces can trap the virus and the viral amount collected when sampling may have been low enough to be detected if the test has low analytic sensitivity [37–39]. Additionally, recovering viruses from porous surfaces can also be limited by the sampling methodology, the recovery media, size of the sampled surface, and the material of the instrument [40].

Exhaust fans and pig pen railing might represent most of the positive samples either with or without viable viruses. This may be explained by the fact that these surfaces are non-porous but also because they are in close proximity, but no direct contact, with the pigs. It is known that PRRSV can become airborne, and thus, it may have landed on railings or exhaust fan cones. Given the levels of dust in pig barns, the virus may have utilized these dust particles as carriers and perhaps assisting it its stability and longevity as fomites, which has been reported for other enveloped viruses such as SARS-CoV-2 [41].

PRRSV strain viability comparisons have been recently reported suggesting that some strains remain viable for longer periods than others which partially explains the successful detection in the present study [13,14,42]. The most common strain present at the farms from which samples were collected was the L1C.5 variant, a virus that emerged in 2021 and became highly prevalence in the U.S. swine population [43,44]. Recently, a study conducted under *in vitro* conditions concluded that this same virus could be detected for up to five hours after contaminating cardboard coupons and maintaining them at 30°C when comparison to other non-porous coupons at the same temperature and time, and to another PRRS MN184 virus [13]. This might explain the current prevalence of L1C.5 in the U.S., but this needs to be explored further as a farm with L1A had similar RNA detections as an L1C.5 positive farm (75%), and the former was the second farm with the most viable particles detected (55%).

For this study, virus isolation was not incorporated was not incorporated due to its limiting factors [45,46] that may yield negative results, especially when the amount of virus is low, which has been found in a previous study involving environmental samples [12]. Therefore, a novel approach for virus viability using RT-qPCR [15] was chosen as the diagnostic tool. The agreement between the detection of RNA using S-RT-qPCR and viable virus by V-RT-qPCR assay from each surface location was determined using Cohen’s kappa. Of the 15 surfaces, three had perfect agreement between the two assays, and the surfaces with only one detection had the lowest agreement (κ = 0.29). This indicates that not all surfaces with positive detections by S-RT-qPCR will contain viable virus. As expected, there were no detections of viable viruses from negative S-RT-qPCR samples. Molecular assays for viability have also been described for *Mycoplasma hyopneumoniae* [47], porcine epidemic diarrhea virus (PEDV) [48], and Influenza A virus (IAV) [49] using different methodologies. Although these types of assays can be limited compared to other tools as they are currently not available to the public, making the use of cell cultures the main option for assessing viability and infectivity which, in certain cases, can underestimate PRRSV viability. Additionally, the cross-sectional sampling approach did not allow to establish the moment when the surface may have been contaminated. This may have played a role in detection as the viral genomic material degraded over time due to environmental factors such as temperature and humidity; however, measuring these variables was not withing the scope of this study. Nonetheless, factors such as these can influence the viability of the virus, as recently reported under experimental settings in which aerosolized, PRRSV has longer half-life at lower temperatures and relative humidity [50]. Similarly, recent reports confirm that PRRS is stable at low temperatures and relative humidity in different surface material types [13,35].

Through the chosen surface selection, sampling methodology, and the use of a novel PRRSV viability RT-qPCR not only the virus’ RNA genome was detected but also intact virus particles. This information creates value for veterinarians and farm staff, specifically those who oversee growing pig herds, by helping them improve and implement PRRS biocontainment, bioexclusion, and biomanagement practices. As this can be used to raise risk awareness and enforce protocol compliance.

## Supporting information

S1 File Data set. (XLSX)
