# Correction: Determining farm surface porcine reproductive and respiratory syndrome virus (PRRSV) contamination through viability RT-qPCR

**DOI:** 10.1371/journal.pone.0352265

**Published:** 2026-06-23

**Authors:** Claudio Marcello Melini, Amanda Palowski, Declan C. Schroeder, Cesar A. Corzo

In Table 3, the percentage values presented in parentheses are incorrect. Please see the correct Table 3 here.

**Table 3:** Number of standard-RT-qPCR and viability-RT-qPCR porcine reproductive and respiratory syndrome virus positive surface samples by material and porosity classification.

Surface material (porosity) classification	RT-qPCR			
	Standard		Viability	
	Positive/total number of samples (%)	Median (Q1, Q3) Ct value	Positive/ total number of samples (%)	Median (Q1, Q3) Ct value
Metal (non-porous)	36/85 (42.4%)	29.2 (28.4, 30.5)	23/85 (27.1%)	28.2 (28.0, 28.5)
Plastic (non-porous)	24/55 (43.6%)	29.1 (28.0, 30.9)	14/55 (25.5%)	27.9 (27.8, 28.1)
Concrete (porous)	12/33 (36.4%)	30.4 (29.7, 32.0)	5/33 (15.2%)	28.3 (27.4, 28.4)
Rubber (non-porous)	3/6 (50.0%)	30.4 (29.2, 32.1)	1/6 (16.7%)	28.2 (28.2, 28.2)
Vinyl (non-porous)	3/8 (37.5%)	27.7 (27.6, 28.1)	3/8 (37.5%)	27.6 (27.4, 27.8)
Glass (non-porous)	2/7 (28.6%)	28.2 (28.1, 28.4)	2/7 (28.6%)	27.2 (27.2, 27.2)
Wood (porous)	0/6 (0%)	- (-, -)	0/6 (0%)	- (-, -)
